# Seed dormancy and germination—emerging mechanisms and new hypotheses

**DOI:** 10.3389/fpls.2014.00233

**Published:** 2014-05-28

**Authors:** Hiroyuki Nonogaki

**Affiliations:** Department of Horticulture, Oregon State UniversityCorvallis, OR, USA

**Keywords:** chromatin remodeling, dormancy, embryo, endosperm, germination, hormone

## Abstract

Seed dormancy has played a significant role in adaptation and evolution of seed plants. While its biological significance is clear, molecular mechanisms underlying seed dormancy induction, maintenance and alleviation still remain elusive. Intensive efforts have been made to investigate gibberellin and abscisic acid metabolism in seeds, which greatly contributed to the current understanding of seed dormancy mechanisms. Other mechanisms, which might be independent of hormones, or specific to the seed dormancy pathway, are also emerging from genetic analysis of “seed dormancy mutants.” These studies suggest that chromatin remodeling through histone ubiquitination, methylation and acetylation, which could lead to transcription elongation or gene silencing, may play a significant role in seed dormancy regulation. Small interfering RNA and/or long non-coding RNA might be a trigger of epigenetic changes at the seed dormancy or germination loci, such as *DELAY OF GERMINATION1*. While new mechanisms are emerging from genetic studies of seed dormancy, novel hypotheses are also generated from seed germination studies with high throughput gene expression analysis. Recent studies on tissue-specific gene expression in tomato and Arabidopsis seeds, which suggested possible “mechanosensing” in the regulatory mechanisms, advanced our understanding of embryo-endosperm interaction and have potential to re-draw the traditional hypotheses or integrate them into a comprehensive scheme. The progress in basic seed science will enable knowledge translation, another frontier of research to be expanded for food and fuel production.

## Introduction

The ultimate role of seeds is to produce offspring and maintain species. Therefore, plants have evolved diverse strategies to ensure successful germination of this genetic delivery system. Proper distribution of seed germination, in both temporal and spatial manners, is critical for survival and proliferation of seed plants. Spatial distribution of germination is generally controlled through seed and fruit morphology, which enhances dispersal of the offspring from the maternal habitat. In contrast, temporal distribution of germination is controlled mainly by the physiological status of seeds. A variation among individual seeds in a population, in terms of physiological status, allows each seed to germinate at a different timing, which is an important strategy for seeds to avoid competition with their siblings or extinction of all individuals due to a disastrous condition. Plants have evolved seed dormancy, temporal suppression of germination under the conditions favorable to germination. Induction of seed dormancy during the maturation stage and its release at a dry state after a certain period of time, which is called “after-ripening,” are widespread phenomena observed in diverse species of seed plants (Bewley et al., 2013). There may be a universal mechanism of seed dormancy as well as a species-specific variation in the regulatory mechanisms.

Hormonal regulation may be a highly conserved mechanism of seed dormancy among seed plants. Induction and maintenance of seed dormancy by abscisic acid (ABA) and dormancy release by gibberellin (GA) are observed in many species. The molecular mechanism of antagonistic function of these two hormones was unclear for many years. However, identification of the rate-limiting hormone metabolism genes, such as nine-*cis*-epoxycarotenoid dioxygenase (*NCED*), an ABA biosynthesis gene and *GA2ox*, a GA deactivation gene, and intensive analysis of their regulatory mechanisms in the last decade, have provided a comprehensive picture of ABA and GA involvement in the seed dormancy mechanisms (Seo et al., 2009). Now, we understand that seed response to light, which varies depending on species, is also controlled through hormone metabolism and signal transduction (Seo et al., 2009). Progress in seed dormancy and germination research is well summarized in recent review articles and textbooks (Graeber et al., 2012; Arc et al., 2013; Bewley et al., 2013). In this review, the main focus will be placed on the most recent discoveries from on-going research of seed dormancy and germination. Therefore, the contents of this review are not meant to be comprehensive but will highlight the “emerging” mechanisms and new hypotheses at the frontier of research.

## Emerging mechanisms of seed dormancy

Previously unknown seed dormancy-associated factors are emerging from on-going research, some of which enhance seed dormancy while others negatively affect it. The positive and negative regulators of seed dormancy, which will be discussed in this section, are summarized in Table 1. There is a risk of over-simplifying gene function with the categorization of positive and negative regulators, because there are complex regulatory mechanisms of seed dormancy, in which a single gene product could exert both positive and negative effects, including negative feedback from a positive regulator. However, to highlight the discoveries of gene function in the original research, this categorization will be used for the discussion in this section.

**Table 1:** **Seed dormancy associated genes described in this article**.

**Symbol**	**Gene name**	**Dormancy function**	**Related publications**
*ABA1*	*ABA deficient 1*	Positive	Bentsink et al., 2006
*ABI3*	*ABA INSENSITIVE 3, 4, 5*	Positive	Zheng et al., 2012
*ABI4*		Positive	Liu et al., 2007
*ABI5*		Positive	Piskurewicz et al., 2008
*ACO1*	*1-AMINOCYCLOPROPANE-1-CARBOXYLATE OXIDASE 1, 4, 5*	Negative	Wang et al., 2013
*ACO4*		Negative	
*ACO5*		Negative	
*AGO4*	*ARGONAUTE 4*	Negative	Singh and Singh, 2012; Singh et al., 2013
*ATXR7*	*ARABIDOPSIS TRITHORAX-RELATED 7*	Positive	Liu et al., 2011
*CYP707A*	*Cytochrome P450 707A*	Negative	Wang et al., 2013
*DEP*	*DESPIERTO*	Positive	Barrero et al., 2010
*DOG1*	*DELAY OF GERMINATION 1*	Positive	Bentsink et al., 2006, 2010; Nakabayashi et al., 2012
*ELF4*	*EARLY FLOWERING 4, 5*	Positive	Liu et al., 2011
*ELF5*		Positive	
*ERF9*	*ETHYLENE RESPONSE FACTOR*	Negative	Wang et al., 2013
*ERF105*		Negative	
*ERF112*		Negative	
*GA3ox*	*GA3-oxidase*	Negative	Yano et al., 2013
*GA2ox*	*GA2-oxidase*	Positive	
*HD2B*	*HISTONE DEACETYLASE 2B, 6, 19*	Negative	
*HDA6*		Positive^*^	Wang et al., 2013
*HDA19*		Positive^*^	
*HDAC1*	*HISTONE DEACETYLATION COMPLEX 1*	Negative^*^	Perrella et al., 2013
*HUB1*	*H2B MONOUBIQUTINATION 1*	Positive	Liu et al., 2007
*KYP*	*KRYPTONITE*	Negative	Zheng et al., 2012
*NCED4*	*NINE-CIS-EPOXYCAROTENOID DIOXYGENASE 4, 9*	Positive	Wang et al., 2013
*NCED9*		Positive	Liu et al., 2007
*PDF1*	*PDF1 protein phosphatase 2A*	Negative	Miatton, 2012
*RDO2*	*REDUCED DORMANCY2* (=*TFIIS*)	Positive	Liu et al., 2011
*RDO4*	*REDUCED DORMANCY2* (=*HUB1*)	Positive	Liu et al., 2007
*Sdr4*	*Seed dormancy 4*	Positive	Sugimoto et al., 2010
*SNL1*	*SIN3-LIKE 1, 2*	Positive	Wang et al., 2013
*SNL2*		Positive	
*SnRK2*	*Snf1-related protein kinase 2*	Positive	Piskurewicz et al., 2008
*SUVH4*	*SU(VAR)3-9 HOMOLOG4 (=KYP)*	Negative	Zheng et al., 2012
*TFIIS*	*Transcription elongation factor S-II*	Positive	Grasser et al., 2009
*VIP7*	*VERNALIZATION INDEPENDENCE 7, 8*	Positive	Liu et al., 2011
*VIP8*		Positive	

* HDA6 and HDA9 are known to affect ABA sensitivity negatively, which could affect seed dormancy negatively.

## Positive regulation

### *DOG1*–central to seed dormancy but unknown for biochemical function

Quantitative trait locus (QTL) analysis using natural variation in Arabidopsis has identified the “seed dormancy-specific” loci, including the *DELAY OF GERMINATION* (*DOG*) genes (Alonso-Blanco et al., 2003; Bentsink et al., 2006, 2010), although some of them might not be strictly specific to dormancy (Chiang et al., 2013). One of them, *DOG1* has been characterized in detail. *DOG1* is expressed in seeds during the maturation stage. Loss of function of *DOG1* results in no dormancy (Bentsink et al., 2006). The genetic role of *DOG1* in seed dormancy and the significance of its expression in environment sensing and adaptation have been well documented (Kronholm et al., 2012; Footitt et al., 2013, 2014).

**Figure 1:**
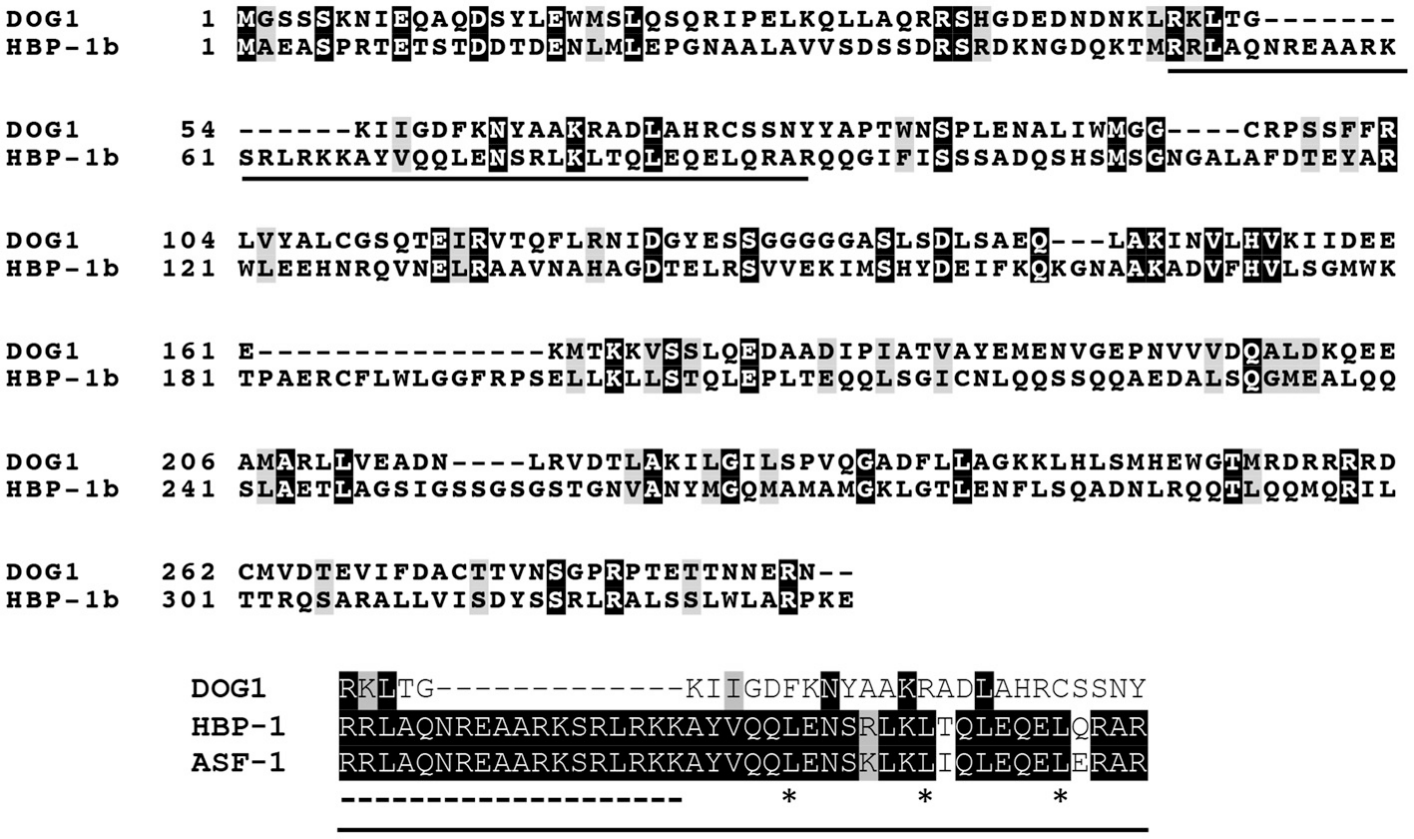
**Alignment of Arabidopsis *DOG1*, wheat HBP-1b and tobacco ASF-1**. Arabidopsis *DOG1* (*DELAY OF GERMINATION1*) encodes an unknown protein, which shows some similarity to the wheat HBP-1b (Histone gene Binding Protein-1b), a leucine zipper class transcription factor (Bentsink et al., 2006). However, the *DOG1* protein does not show high identity to the leucine zipper domain in the HBP-1b (solid underline in the top panel). This region (extracted as the bottom panel) contains the basic motifs (dashed underline) and the heptad-repeat leucines (asterisks) in the leucine zipper structure (Tabata et al., 1991), which are conserved among the wheat HBP-1b, tobacco ASF-1 (Activation Sequence Factor-1) and other leucine zipper transcription factors. Sequences were aligned using the ClustralW and boxshade programs (http://www.expasy.org/genomics/sequence_alignment).

In contrast, the biochemical and molecular function of *DOG1* is still a mystery. *DOG1* encodes an unknown protein, for which only limited information is available. The *DOG1* cDNA shows highest similarity with a *Brassica napus* EST from an embryo library, however this gene also is not annotated. The protein with a known function that shows the highest similarity with *DOG1* is the wheat transcription factor Histone gene Binding Protein-1b (HBP-1b) (Bentsink et al., 2006). HBP-1b is a leucine zipper class transcription factor, which binds to the H3 hexamer motif ACGTCA in the promoter regions of wheat histone H3 genes (Mikami et al., 1989). This motif is required for transcription of the wheat H3 histone gene (Nakayama et al., 1989). *DOG1* has also been suggested to be a transcription factor, which is supported by its localization in the nucleus (Nakabayashi et al., 2012). However, the identity between *DOG1* and HBP-1b is not very high especially in the basic motifs and the heptad-repeat leucines in the leucine zipper structure (Tabata et al., 1991), which are conserved in HBP-1b and other H3 hexamer-binding proteins, such as tobacco Activation Sequence Factor-1 (ASF-1) (Lam et al., 1989) (Figure 1). Therefore, the biochemical function of *DOG1* is hardly predicted from its moderate similarity to HBP-1b. So far, direct target genes of *DOG1* that are clearly linked to the seed dormancy mechanisms have not been identified, although some dormancy up-(Dup) regulated genes [e.g., At5g43580 (*PR peptide*), At5g45540 (*unknown protein*), At5g45830 (*DOG1*), At5g47160 (*YDG/SRA domain-containing protein*)] or dormancy down-(Ddown) regulated genes [At4g19700 (*E3 ubiquitin ligase*), At5g04220 (*SYNAPTOTAGMIN3*), At5g46160 (*ribosomal protein*)] in the *DOG1* near isogenic line (NIL) have been identified (Bentsink et al., 2010).

### Possible modification and partners of *DOG1*

*DOG1* transcript accumulates during the seed maturation stage with its peak around 14–16 days after pollination (DAP) (Bentsink et al., 2006), is reduced to about 20% in freshly harvested seeds, and disappears during imbibition (Nakabayashi et al., 2012). *DOG1* protein also accumulates during the maturation stage, however the protein level does not decrease toward the completion of seed maturation. As a consequence, freshly harvested seeds contain a relatively high level of *DOG1* protein. The protein level still remains relatively high even after 13 weeks of after-ripening when seed dormancy is already released (Nakabayashi et al., 2012). Thus, a correlation is lacking between the amount of *DOG1* protein and dormancy levels in after-ripened seeds. It has been proposed that the chemical property of *DOG1* protein, rather than its amount, is critical for *DOG1* to maintain seed dormancy and that its alteration to a non-functional form during after-ripening allows seed germination (Nakabayashi et al., 2012). In fact, there is a shift in the pI (isoelectric point) of the *DOG1* peptides prior to and following after-ripening (Nakabayashi et al., 2012).

Induction of *DOG1* in imbibed *dog1* mutant seeds with a heat-shock inducible system does not cause dormancy and allows 100% germination (Nakabayashi et al., 2012). This can be explained by the lack of protein modification discussed above. When *ABI5*, another key dormancy gene was overexpressed in Arabidopsis seeds, it was not sufficient to suppress germination. Only when the SnRK2 (Snf1-related protein kinase2), which activates ABI5, was induced in imbibed seeds, ABI5 was able to suppress seed germination (Piskurewicz et al., 2008). Therefore, it is possible that the *DOG1* protein induced by the heat-shock system was missing necessary modification in the ectopic induction experiment.

Recently, a search for possible *DOG1* partners was conducted through a yeast two-hybrid screen, which identified multiple proteins, including the PDF1 protein phosphatase 2A (Miatton, 2012). *PDF1* expression is enriched in the vascular system of the embryo (Miatton, 2012), which mimics the *DOG1* localization (Nakabayashi et al., 2012). PDF expression has its peak around 16 DAP during the maturation stage and is reduced in mature seeds, which is similar to the *DOG1* expression mentioned above. Unlike the *dog1* mutant, the *pdf1* loss of function mutant exhibits an enhanced seed dormancy phenotype (Miatton, 2012), suggesting that PDF1 is a negative regulator of seed dormancy and antagonizes *DOG1*. It is hypothesized that *DOG1* requires phosphorylation to be active, in terms of its function in seed dormancy induction and maintenance, and is dephosphorylated by PDF1, which could inactivate *DOG1* (Miatton, 2012). More analysis of PDF1 and other *DOG1*-interacting proteins will potentially provide a breakthrough in seed dormancy research.

Regardless of posttranslational modification, an alternative hypothesis to explain the lack of seed dormancy in *DOG1*-induced *dog1* seeds is that *DOG1* functions mainly during the maturation stage and the *DOG1* protein contained in mature seeds might be residual. It is possible that *DOG1* affects seed dormancy through its effects on ABA levels during maturation (Nakabayashi et al., 2012). *DOG1* has been proposed to function in a pathway independent of plant hormones. However, *DOG1* is not able to impose seed dormancy in *aba1-1*, an ABA-deficient mutant (Bentsink et al., 2006), indicating that *DOG1* function is dependent on ABA. ABA levels are reduced in *dog1* mutants while GA levels are enhanced (Bentsink et al., 2006; Nakabayashi et al., 2012), supporting the idea of possible links between the *DOG1* and hormone pathways in seed dormancy. More information is necessary to obtain a clear picture about the hormone dependent and independent pathways of seed dormancy. To date, induction of *DOG1* specifically at the right timing during seed maturation (14–16 DAP) has not been experimentally examined. Investigation of molecular consequences upon *DOG1* induction at the right timing, including gene expression, protein phosphorylation and epigenetic changes (discussed below), will provide useful information. It should be noted that there are other dormancy(-specific) genes recently discovered, such as *Seed dormancy 4* (*Sdr4*) in rice (Sugimoto et al., 2010) and *DESPIERTO* in Arabidopsis (Barrero et al., 2010), which were not discussed here. Those genes also appear to be central to the dormancy mechanisms and are important targets of seed dormancy research.

### Transcription elongation of seed dormancy genes

**Figure 2:**
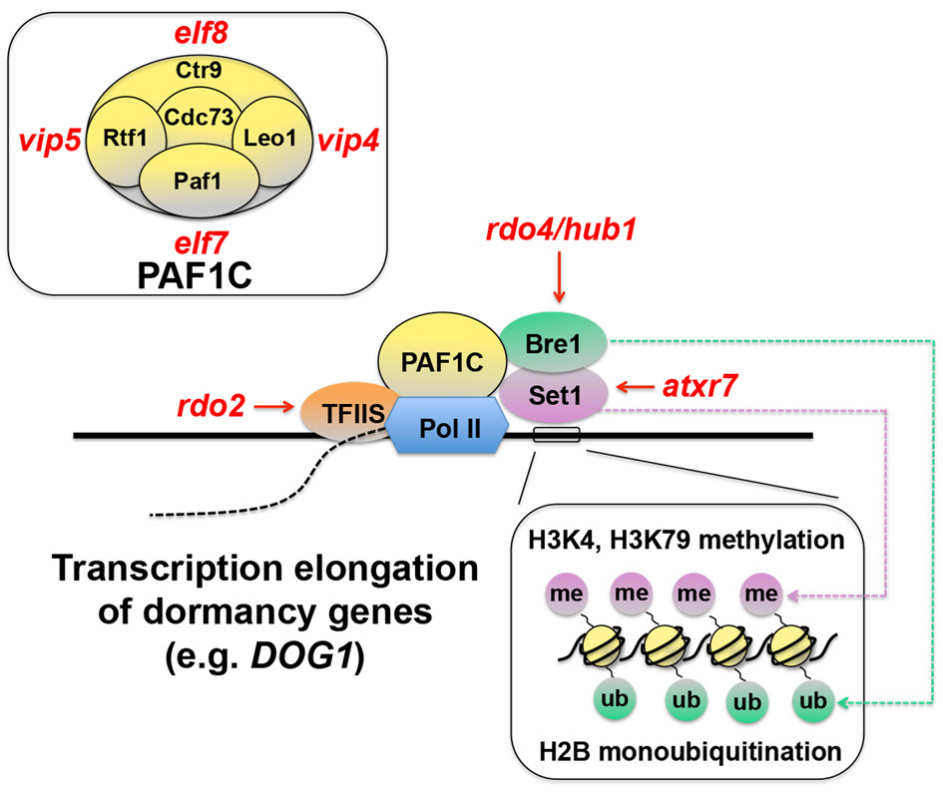
**Schematic representation of transcription elongation of seed dormancy genes**. Transcription elongation factor S-II (TFIIS) assists RNA polymerase II (Pol II) and promotes transcription elongation (Saunders et al., 2006; Kim et al., 2010). Pol II-Associated Factor 1 Complex (PAF1C), which consists of Paf1, Rtf1, Ctr9, Leo1, and Cdc73 (top-left inset) in yeast (Porter et al., 2005), also functions in this process through its interaction with Bre1, which monoubiquitinates (ub) histone 2B (H2B), and Set1, which methylates (me) histone H3 lysine 4 (H3K4), and lysine 79 (H3K79) (bottom-right inset) (Sun and Allis, 2002; Zhu et al., 2005; Kim et al., 2009). These chromatin-remodeling events and their positive effects on transcription elongation are thought to be critical for induction of seed dormancy genes, because mutants in many of these components (*rdo2, rdo4, atxr7, elf 7, elf8, vip4*, and *vip5*) exhibit reduced seed dormancy (Liu et al., 2011). Red italic symbols indicate Arabidopsis mutants corresponding to the yeast protein components. *atxr7, arabidopsis trithorax-related 7; elf, early flowering*; *hub1, h2b monoubiquitination1*; *rdo, reduced dormancy*; *vip, vernalization independence*.

There is emerging evidence to suggest that regulation of transcriptional efficiency may be one of the core mechanisms of seed dormancy. Transcriptional efficiency is determined by recruitment of RNA polymerase II (Pol II) to the DNA template and the rate of transcription elongation after its binding to DNA. The efficiency of transcription elongation is influenced by an arrest of Pol II and its recovery from the arrest (Saunders et al., 2006). Transcription elongation factor S-II (TFIIS) assists Pol II to overcome the temporal arrest during elongation and enhances RNA synthesis (Kim et al., 2010) (Figure 2). A mutagenesis screen for seed dormancy in Arabidopsis yielded *reduced dormancy* (*rdo*) mutants (Leon-Kloosterziel et al., 1996; Peeters et al., 2002). *RDO2*, one of the genes identified from this screening, encoded TFIIS (Liu et al., 2011). Another independent study also found that a mutation in *TFIIS* resulted in reduced seed dormancy (Grasser et al., 2009). These results suggest that transcription elongation may be a critical part of the dormancy mechanisms.

The phenotypes of other mutants also support this contention. TFIIS and Pol II interact with the Pol II-Associated Factor 1 Complex (PAF1C) (Kim et al., 2010) (Figure 2). In yeast, PAF1C consists of Paf1, Rtf1, Ctr9, Leo1, and Cdc73 (Penheiter et al., 2005; Porter et al., 2005) (Figure 2, top-left inset). The Arabidopsis orthologs of these yeast proteins EARLY FLOWERING7 (ELF7) (= Paf1), ELF8 (= Ctr9), VERNALIZATION INDEPENDENCE4 (VIP4) (=Leo1), VIP5 (= Rtf1) and PLANT HOMOLOGOUS TO PARAFIBROMIN (PHP) (= Cdc73) have been identified (Zhang and Van Nocker, 2002; He et al., 2004; Oh et al., 2004; Yu and Michaels, 2010). Seeds of the *elf7, elf8, vip4*, and *vip5* mutants all exhibit reduced dormancy (Liu et al., 2011), suggesting the importance of PAF1C and transcription elongation for seed dormancy.

### Histone ubiquitination and methylation associated with transcription elongation

PAF1C interacts with Bre1, a protein involved in histone 2B (H2B) monoubiquitination (Kim et al., 2009) (Figure 2). Interestingly, *rdo4*, another reduced dormancy mutant in Arabidopsis, which was isolated from the same mutagenesis screening as mentioned above, has a mutation in *H2B MONOUBIQUITINATION1* (*HUB1*) gene, an Arabidopsis ortholog of *Bre1* (Liu et al., 2007). Bre1 interacts with Set1, which methylates histone 3 lysine 4 and lysine 79 (H3K4, H3K79) (Sun and Allis, 2002; Zhu et al., 2005) and promotes gene expression (Figure 2). A mutation in the *Set1* ortholog *ARABIDOPSIS TRITHORAX-RELATED 7* (*ATXR7*) also causes reduced dormancy in seeds (Liu et al., 2011). These results reinforce the idea that regulation of transcription elongation efficiency is an essential part of seed dormancy and suggest the significance of chromatin remodeling in the regulatory mechanisms.

H2B monoubiquitination and H3K4 and H3K79 methylation, which is dependent on H2B monoubiquitination (Nakanishi et al., 2009), are thought to activate gene expression (Henry et al., 2003). Since *hub1* (=*bre1*) seeds exhibit reduced dormancy, genes down-regulated in the *hub1* mutant are good candidates for seed dormancy-imposing genes, the expression of which is promoted through transcriptional elongation. *ABA INSENSITIIVE4* (*ABI4*), *DOG1, NINE-CIS-EPOXYCAROTENOID DIOXYGENASE9* (*NCED9*) and other genes have been identified as possible targets of HUB1/RDO4 (Liu et al., 2007). *RDO2* (*TFIIS*) and *RDO4* (*HUB1*), two positive regulators of transcription are induced during the same stages of seed maturation (~18–19 DAP). There is a significant overlap between *rdo2* and *rdo4*, in terms of differentially expressed genes in the mutants. These results suggest that RDO2 and RDO4 might share common targets. Intriguingly, *DOG1* is one of the genes commonly down-regulated in the two mutants (Liu et al., 2011). Activation of *DOG1* through chromatin remodeling and transcriptional elongation might be an important mechanism of seed dormancy.

The hypothesis that seed dormancy is regulated by the efficiency of transcription elongation of *DOG1* is also supported by the recent analysis of the *tfIIs* mutant, in which seed dormancy is reduced but reverted to the wild-type level by an extra copy of *DOG1* (Mortensen and Grasser, 2014). However, when the *hub1/rdo4* mutant is crossed with the NIL carrying *DOG1*-Cvi, which causes deep seed dormancy, the resulting seeds still show dormancy at a level between *hub1* and *DOG1*-Cvi NIL. Similar results are observed when the *hub1/rdo4* was transformed with the Cvi *DOG1* genomic fragment. The incomplete alleviation of dormancy from NIL *DOG1* by *hub1/rdo4* mutation in both cases suggests that *HUB1* is not epistatic to *DOG1.* In contrast, the combination of *hub1* and *DOG3*-Cvi resulted in no seed dormancy, suggesting that *HUB1* functions in the same pathway as *DOG3* to affect seed dormancy (Liu et al., 2007). More analyses of the specific targets of epigenetic modification and transcriptional elongation will be necessary to draw a clear picture about seed dormancy regulation through these processes.

### Repression of seed germination genes through histone deacetylation

**Figure 3:**
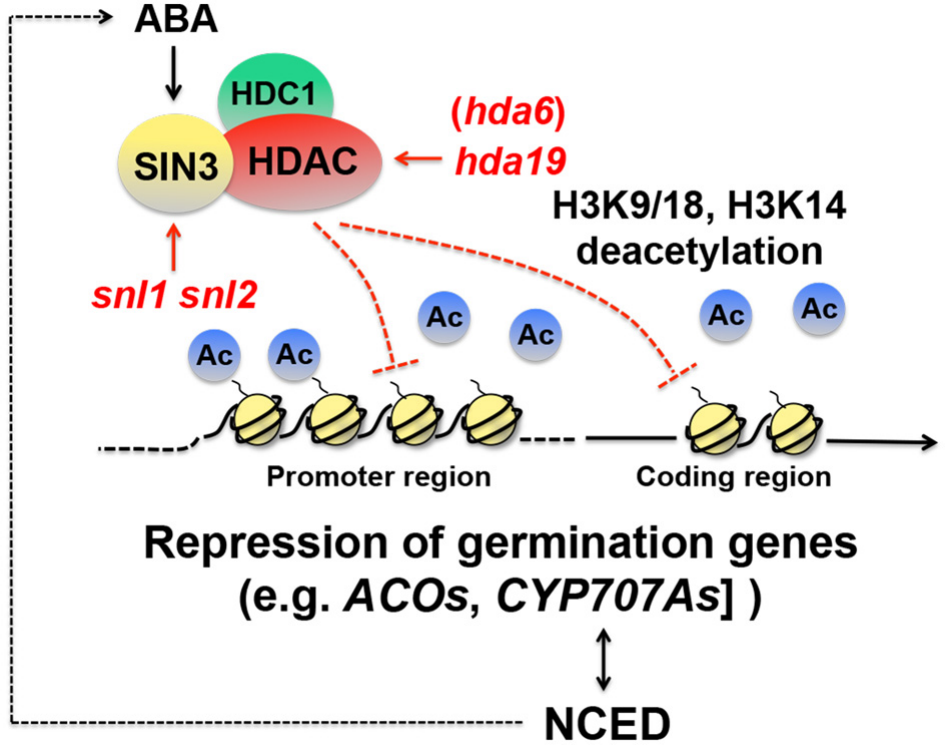
**Schematic representation of repression of seed germination genes through histone deacetylation**. In yeast, a histone deacetylase (HDAC) interacts with SWI-INDEPENDENT3 (SIN3) (Kadosh and Struhl, 1998; Lai et al., 2001; Grzenda et al., 2009). HDA19, an HDAC ortholog in Arabidopsis, interacts with SIN3-LIKEs (SNLs) (Wang et al., 2013) and HDC1 (Histone Deacetylation Complex1) (Perrella et al., 2013), and removes acetyl groups (Ac) from histone 3 lysine9/18 (H3K9/18) and lysine14 (H3K14) and represses genes positively affecting germination, such as *1-AMINOCYCLOPROPANE-1-CARBOXYLATE OXIDASEs* (*ACOs*) and *CYP707As*, ABA deactivation genes (Wang et al., 2013). Deacetylation occurs in both the promoter and coding regions. Both *snl* and *hda19* mutations cause reduced dormancy (Wang et al., 2013). Expression of *NCED4*, an ABA biosynthesis gene, is reduced in the *snl1 snl 2* double mutant, suggesting that the SNL-HDA19 complex imposes seed dormancy also through the promotion of ABA biosynthesis. *SNL* expression is promoted by ABA (Wang et al., 2013), which suggests that there is a positive feedback loop to maintain high ABA levels through the SNL-HDA19 pathway.

While activation of dormancy genes through transcription elongation appears to be critical for dormancy induction, continuous repression of seed germination-associated genes is also probably an essential part of dormancy maintenance. There is evidence that histone deacetylation is imperative for repression of genes positively affecting seed germination. In yeast and mammals, histone deacetylase (HDAC) interacts with SWI-INDEPENDENT3 (SIN3), an amphipathic helix repeat protein, removes acetyl groups from lysine in the histone tails, and creates a transcriptionally inactive state of chromatin (Kadosh and Struhl, 1998; Lai et al., 2001; Grzenda et al., 2009) (Figure 3). In Arabidopsis, SIN3-LIKE1 (SNL1) physically interacts with HDA19, an Arabidopsis HDAC ortholog, both *in vitro* and *in planta* (Wang et al., 2013). The Arabidopsis genome contains *SNL2*, which is partially redundant to *SNL1*. Seeds of the *snl1 snl2* double mutant exhibit reduced dormancy. A reduced dormancy phenotype is also observed in *hda19* mutant seeds (Wang et al., 2013). These results indicate that SNLs and HDA19 are positive regulators of seed dormancy. It appears that proper repression of the SNL-HDA19 targets, which are most likely germination-inducing genes, through histone deacetylation is essential for normal seed dormancy. Acetylation of H3K9/18 and H3K14 is increased in the *snl1 snl2* double mutant (Wang et al., 2013), which confirms that in wild-type seeds the SIN3-HDAC complex deacetylates histones and puts repressive marks on the chromatin (Richon and O'Brien, 2002) (Figure 3).

Global gene expression analysis between the *snl1 snl2* double mutant and wild-type seeds with RNA sequencing identified possible targets of SNL-HDA19. Ethylene biosynthesis genes, such as *1-AMINOCYCLOPROPANE-1-CARBOXYLATE OXIDASE1* (*ACO1*), *ACO4*, and *ACO5* and ethylene response genes, such as *ETHYLENE RESPONSE FACTOR9* (*ERF9*), *ERF105*, and *ERF112*, were up-regulated in the mutant (Wang et al., 2013). Quantitative PCR combined with chromatin immunoprecipitation with the H3K9/18 acetylation-specific antibodies showed that the *ACOs* and *ERFs* genes were indeed hyperacetylated in the mutant, which mainly occurred in the promoter region but were also found in the coding region (Wang et al., 2013). These results suggest that SNL-HDA19 causes seed dormancy by suppressing the ethylene pathway, which positively affects seed germination in Arabidopsis (Chiwocha et al., 2005; Arc et al., 2013).

In contrast, the same study suggests that SNL-HDA19 increases ABA levels and thereby enhances seed dormancy. *CYP707A1* and *CYP707A2*, ABA deactivation genes, which reduce ABA levels, were up-regulated in the *snl1 snl2* double mutants. Consistently, *NCED4*, an ABA biosynthesis gene, was down-regulated in the same mutant (Wang et al., 2013). These results suggest that SNL-HDA19 suppresses *CYP707As* and activates *NCED4* in wild type, both of which increase ABA levels and enhance seed dormancy. Interestingly, ABA stimulates *SNL1* and *SNL2* expression (Wang et al., 2013), which suggests that there is positive feedback regulation to maintain high levels of ABA through the histone deacetylation pathway (Figure 3). While this study suggests that ABA levels are positively affected by SNL-HDA19, other studies suggest that ABA sensitivity is negatively regulated by HDA19 (and HDA6). Mutations in *HDA6* and *HDA19* cause ABA hypersensitivity during germination (Chen et al., 2010; Chen and Wu, 2010). Loss of function in Histone Deacetylation Complex1 (HDC1), another component of the SNL- and HDA19-containing complex, which physically interacts with HDA6 and HDA19 (Figure 3), also causes ABA hypersensitivity in seedlings. *HDC1* overexpression promotes seedling emergence (Perrella et al., 2013), although detailed information about *sensu stricto* germination and a dormancy phenotype of the mutant seeds is not available. The significance of the opposite effects of the HDAC multiprotein complex to ABA levels (positive) and sensitivity (negative) in the regulatory mechanisms of seed dormancy is not known. It is possible that the seemingly counterintuitive effects are associated with negative feedback regulation.

## Negative regulation

### Repression of dormancy genes and activation of germination genes through histone deacetylation

*HISTONE DEACETYLASE 2B* (*HD2B*), another *HDAC* gene, is also involved in seed dormancy. In this case, it negatively affects seed dormancy (Yano et al., 2013). This discovery was made through a combination of genome-wide association mapping (GWA) (Atwell et al., 2010) and transcriptomics. The efficiency of QTL analysis using different accessions of Arabidopsis, such as Cvi, L*er*, and Col, for seed dormancy is well exemplified by the successful identification and characterization of the *DOG* genes (Alonso-Blanco et al., 2003; Bentsink et al., 2006, 2010). Since the comparison of a few different Arabidopsis accessions is so powerful, multiplying this approach using many accessions with natural variations in seed dormancy is expected to produce fruitful outcomes in seed dormancy research, especially when it is combined with GWA, which identified a number of single nucleotide polymorphisms (SNPs) likely associated with various phenotypes (Atwell et al., 2010). Based on this concept, 113 accessions were analyzed to identify SNPs associated with natural variation in seed dormancy using GWA and transcriptomics, which identified *HD2B* as a strong candidate of a seed dormancy-associated gene. *HD2B* expression levels are significantly lower in 24 dormant accessions than 28 less-dormant accessions, although there are some exceptions. When the highly dormant Cvi line was transformed with the genomic fragment of Col *HD2B* (termed ^Col^*HD2B*/Cvi), mature seeds of ^Col^*HD2B*/Cvi exhibited reduced dormancy, which was not evident immediately after harvest without cold stratification but became clear when seeds were stratified or partially after-ripened (Yano et al., 2013).

Cold stratification releases seed dormancy through an increase in GA levels. *GA3ox1*, a rate-limiting GA biosynthesis gene, is induced by cold stratification (Yamauchi et al., 2004), which triggers expansion of cortex cells in the radicle/hypocotyl region and then generates growth potential of the embryo for germination (Ogawa et al., 2003). Evidence suggests that HD2B mediates this dormancy-releasing process. In ^Col^*HD2B*/Cvi seeds, expression of *GA3ox1* and *GA3ox2* and GA_4_ levels are increased, while expression of *GA2ox2*, a GA deactivation gene, is reduced compared to wild-type Cvi seeds (Yano et al., 2013). Since HDAC represses gene expression through histone deacetylation, *GA2ox2* repression could be a direct effect of HD2B. In contrast, the up-regulation of *GA3ox* genes may be through repression of their upstream regulators or some other mechanisms. It is interesting that the three separate hormone pathways (ethylene, ABA, and GA) associated with seed dormancy are regulated by histone deacetylation. These results demonstrate that epigenetic regulation through chromatin remodeling is a robust mechanism to alter hormone levels in seeds.

### Silencing of seed dormancy genes through histone and DNA methylation

**Figure 4:**
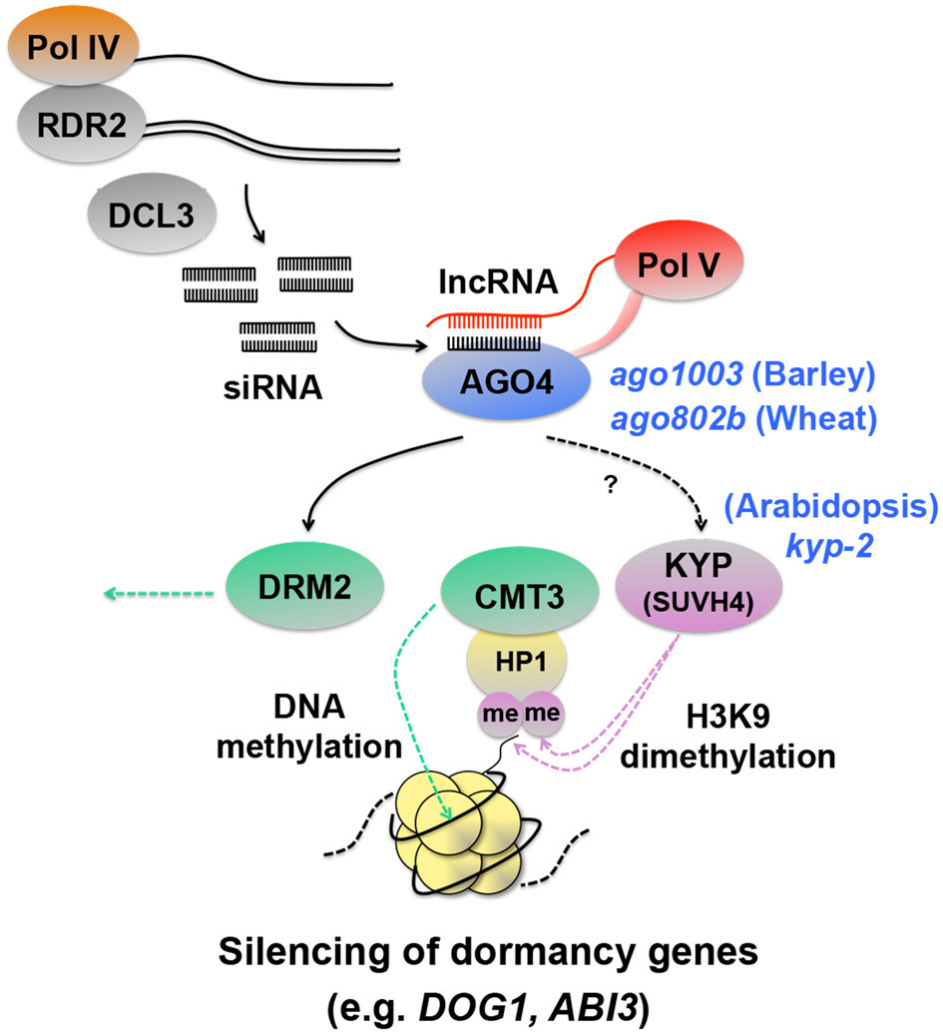
**Schematic representation of silencing of dormancy genes through histone and DNA methylation**. RNA polymerase IV (Pol IV) transcripts are converted to double-stranded RNA by RNA-Dependent RNA polymerase 2 (RDR2), which are then processed into 24-nt siRNAs by DICER-LIKE3 (DCL3) (Xie et al., 2004; Herr et al., 2005; Onodera et al., 2005; Law et al., 2011). siRNAs are loaded onto ARGONAUTE4 (AGO4) (Qi et al., 2006) and interact with long non-coding RNAs (lncRNAs) produced by Pol V, which are thought to function as scaffold transcripts to guide siRNAs to specific loci to be silenced (Wierzbicki et al., 2008, 2009; Wierzbicki, 2012). In this way, the AGO4 complex containing siRNAs and lncRNAs triggers RNA-directed DNA methylation (RdDM) (Wierzbicki, 2012). A possible event downstream of AGO4 is histone 3 lysine 9 dimethylation (H3K9me2) by the KRYPTONITE (KYP), which causes HETEROCHROMATIN PROTEIN1 (HP1) to bind to the modified histone and recruit CHROMOMETHYLASE3 (CMT3), a DNA methyltransferase that induces gene silencing (Jackson et al., 2002; Zilberman et al., 2004; Tran et al., 2005; Johnson et al., 2007). A mutation in *KYP* in Arabidopsis causes enhanced dormancy and up-regulation of *DOG1* and *ABI3* (Zheng et al., 2012), suggesting that the seed dormancy genes are silenced by the KYP-CMT3 pathway. The AGO4 complex is also involved in gene silencing by DOMAINS REARRANGED METHYLTRANSFERASE2 (DRM2) (Zilberman et al., 2004; Wierzbicki, 2012), although DRM2 involvement in seed dormancy regulation is not known. Direct evidence for siRNAs and lncRNAs involvement in *DOG1* and *ABI3* regulation is lacking, however AGO4 has been shown to be a negative regulator of dormancy in barley and wheat seeds (Singh and Singh, 2012; Singh et al., 2013). The Arabidopsis, barley and wheat seed dormancy mutants corresponding to the protein components in the RdDM pathway are indicated by blue italic symbols.

The studies mentioned above showed that HDAC could affect seed dormancy either positively (HDA19) or negatively (HD2B), depending on the target genes. Histone methylation also affects seed dormancy in both ways. While H3K4 and H3K79 methylation activates gene expression and causes seed dormancy as mentioned above (Set1 or ATXR7), dimethylation of H3K9 (H3K9me2), a repressive mark, occurs on the chromatin associated with seed dormancy genes. Analysis of gene silencing at the Arabidopsis *SUPERMAN* (*SUP*) locus identified the KRYPTONITE (KYP) methyltransferase, which causes H3K9me2 (Figure 4). The methylated histone recruits the DNA methyltrasferase CHROMOMETHYLASE3 (CMT3) through its interaction with HETEROCHROMATIN PROTEIN1 (HP1) and triggers the methylation of cytosine nucleotides of DNA and silences the gene (Jackson et al., 2002; Johnson et al., 2007) (Figure 4). KYP is SU(VAR)3-9 (Rea et al., 2000) HOMOLOG 4 and is also called SUVH4. The *kyp-2* mutant seeds show enhanced dormancy, suggesting that KYP/SUVH4 suppresses seed dormancy genes. Interestingly, again, *DOG1* is one of the up-regulated genes in the mutant, as well as *ABI3* (Zheng et al., 2012). These results suggest that histone methylation caused by KYP/SUVH4 induces silencing of *DOG1* and *ABI3* through DNA methylation and negatively affects seed dormancy.

The KYP-CMT3 gene-silencing pathway mediates RNA-directed DNA methylation (RdDM), which is triggered by small interfering RNAs (siRNAs) produced by DICER-LIKE3 (DCL3) and their loading onto ARGONAUTE4 (AGO4) (Zilberman et al., 2004; Tran et al., 2005) (Figure 4). AGO proteins are components of the RNA-induced silencing complex (RISC) and are involved in gene silencing. While AGO1 and AGO10 proteins function mainly in posttranscriptional gene silencing (PTGS) through the MIR (microRNA) and TAS (trans-acting siRNA) pathways, the AGO4/AGO6/AGO9 clade proteins are associated with transcriptional gene silencing (TGS) through RdDM (Mallory and Vaucheret, 2010). Little information is available for silencing of seed dormancy genes through RdDM, however a possible involvement of AGO4 in seed dormancy regulation has been suggested from studies of cereal seed dormancy. *AGO1003*, an *ARGONAUTE* (*AGO*)*4_9* gene in barley, is expressed differentially in the embryos of dormant and non-dormant grains and is thought to function as a negative regulator of seed dormancy through RdDM (Singh and Singh, 2012). A separate study in wheat supports this hypothesis. *AGO802B*, a wheat ortholog *of AGO4_9* gene is expressed during grain development (5–20 DAP). *AGO802B* expression is significantly lower in preharvest sprouting (PHS)-resistant (i.e., more dormant) varieties than in susceptible ones (Singh et al., 2013). This result also suggests that *AGO4* is a negative regulator of dormancy. It is not known whether specific coding genes are subjected to silencing through RdDM in wheat seeds. However, analysis of 5S ribosomal DNA from PHS-resistant and susceptible varieties with the methylation-sensitive restriction enzyme Msp*I* suggested that ribosomal DNA methylation was reduced in PHS-resistant varieties (Singh et al., 2013), supporting the hypothesis that AGO4 enhances histone and DNA methylation and acts as a negative regulator of seed dormancy.

**Figure 5:**
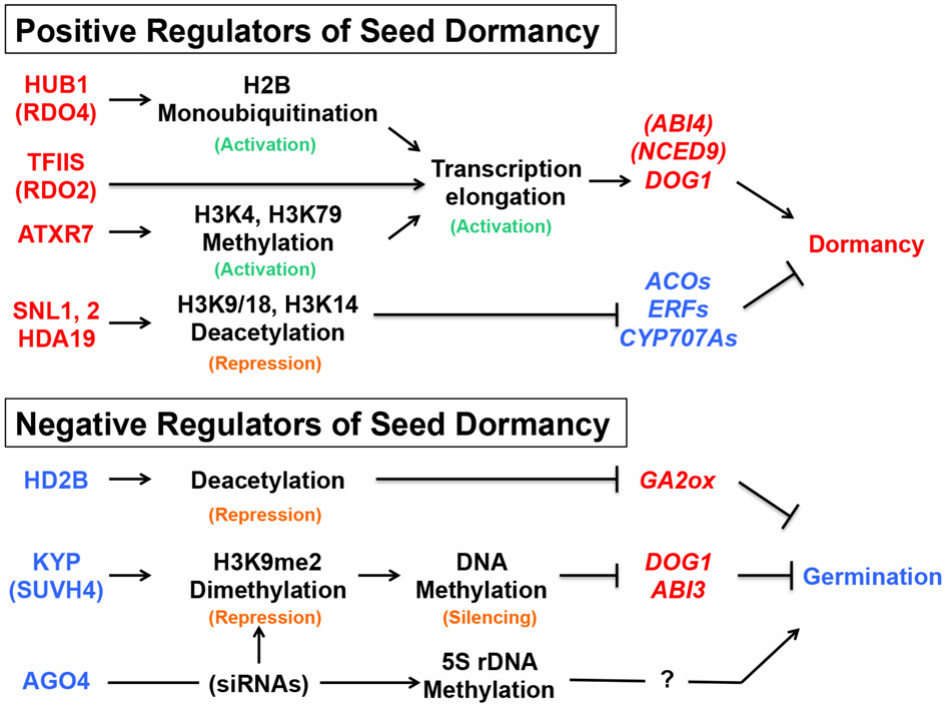
**Summary of the seed dormancy or germination pathways described in this article**. The positive (red) and negative (blue) regulators of seed dormancy and their roles in the chromatin remodeling, DNA modification or siRNA pathways are indicated, together with promotive (arrows) or suppressive (blocked arrows) effects on the downstream genes (italics). Active (green) or repressive (orange) marks on histones or DNA are also indicated. See text for gene and protein symbols and references.

The chromatin-remodeling factors mentioned above include both positive and negative regulators of seed dormancy, which could be considered as negative and positive regulators of seed germination, respectively. The description “activation of seed dormancy genes” or “repression of seed germination genes,” which could mean the same consequence (dormancy or no germination), is confusing. It is even more confusing when the description is combined with different terminology of histone modification, such as histone (de)acetylation, monoubiquitination or (de)methylation, because they could be either repressive or active marks depending on the position of residues in the histone tail. To avoid the confusion, the positive and negative regulators of seed dormancy, their roles in chromatin and DNA modification, and possible consequences in gene expression downstream are summarized in Figure 5.

## New hypotheses for germination events

### Remaining barriers of seed germination

A quiescent state of the embryo is changed when molecular repression on seed germination genes is removed, which is probably orchestrated with silencing of dormancy genes. However, an active embryo is still unable to complete germination when the suppressive force, or mechanical resistance, of the covering tissues, such as the testa and endosperm, exceeds embryo growth potential. When the embryo is not dormant, it is the mechanical resistance of the covering tissues that mainly determines whether the embryo emerges from imbibed seeds. In fact, the embryos in dormant seeds in many species are able to grow when they are excised from seeds, which is called coat-imposed dormancy (Bewley et al., 2013). While further increase in embryo growth potential may still be necessary, alteration of the properties of covering tissues plays a significant role in germination. The testa in a mature seed is generally a non-living tissue, therefore the major reduction in the mechanical resistance of the covering tissues depends on physiological changes in the living endosperm. Changes in the properties of the endosperm significantly affect timing of radicle emergence in non-dormant seeds also. Therefore, the mechanisms of endosperm weakening have been a focal point in seed germination research.

**Figure 6:**
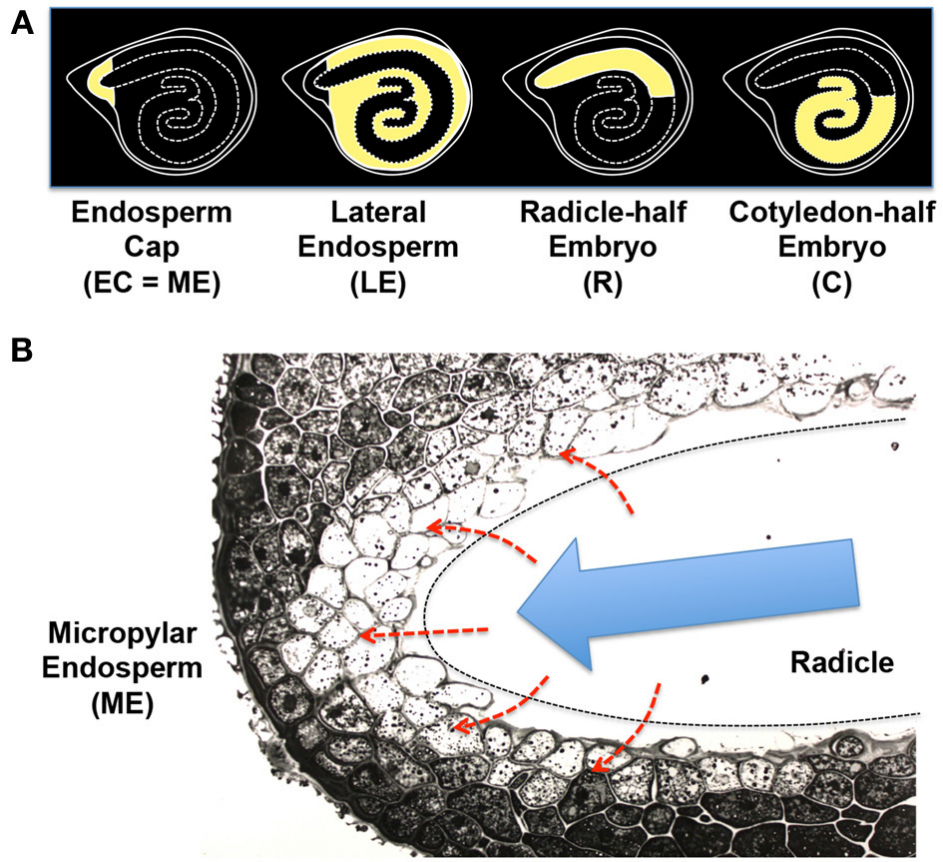
**Tomato seed tissues and regulatory mechanisms of endosperm weakening. (A)** Schematic representation of the four different tissue parts of tomato seeds used for the transcriptome analysis (Martinez-Andujar et al., 2012). Endosperm cap [EC = ME (micropylar endosperm)], lateral endosperm (LE), radicle-half embryo (R), and cotyledon-half embryo (C) are highlighted by yellow filling. **(B)** Photograph of micropylar endosperm (ME) cells. The radicle tip (outlined by dashed line) has been removed. Note that cell walls are fuzzy and protein storage vacuoles and lipid bodies are disappearing in the inner layers of ME cells facing the embryo (Nonogaki et al., 1998). Possible secretion of GA or peptides from the embryo to the endosperm (red dashed arrows) and the growth potential generated by the embryo (blue arrow), which provides pressure onto the endosperm, are indicated in the scheme.

Basic information about endosperm weakening is summarized in other literature (Linkies et al., 2010; Bewley et al., 2013). Briefly, the micropylar region of endosperm (ME) surrounds the radicle tip and provides an opposing force to it (Figure 6), which is reduced during germination through weakening. The mechanical resistance of ME is mainly due to the thick and rigid cell walls in this tissue. Therefore, cell wall modification is thought to play an essential role in ME weakening (Bewley et al., 2013). In fact, genes encoding cell wall-modifying proteins, such as xyloglucan endotransglycosylase/hydrolases (XTHs) and expansins (EXPs), are expressed exclusively in ME of Arabidopsis (Dekkers et al., 2013), *Lepidium sativum* (Voegele et al., 2011) and tomato (Chen and Bradford, 2000; Chen et al., 2002) seeds during germination. While distinct cell wall architecture is observed in ME of seeds depending on plant species and family (Lee et al., 2012a), ME weakening by cell wall modifying proteins seems to be a widely conserved mechanism of germination.

### Embryo-endosperm interaction in tomato seeds

A high throughput transcriptome analysis of germinating tomato seeds showed enrichment of cell wall-associated genes in ME (Martinez-Andujar et al., 2012), supporting the hypothesis discussed above. In this study, tomato seeds were dissected into the endosperm cap (EC, equivalent to ME), lateral endosperm (LE), radicle-half embryo (R), and cotyledon-half embryo (C) (Figure 6A). In addition to the cell wall-associated genes, PR (pathogenesis-related) or wound-response genes were detected as another major group of ME-enriched genes. The 5' upstream sequences of the ME-enriched PR genes contain the conserved sequences, including the DNA motifs targeted by ethylene response factors (ERFs). Interestingly, *Tomato ERF1* (*TERF1*), an experimentally validated upstream regulator of the PR genes, was also one of the ME-enriched genes in tomato seeds (Martinez-Andujar et al., 2012). These results suggest that TERF1 is a major upstream regulator in ME and induces other ME genes, such as PR- or wound response genes and possibly cell wall-associated genes also.

The degradation of cell wall in ME of tomato seeds, which is accompanied by disappearance of storage vacuoles and lipid bodies from the cells, is initiated at the inner cells adjacent to the radicle tips (Figure 6B), suggesting that ME activation is under the control of the embryo. A traditional view of the mechanism of ME gene induction is that diffusible signals, such as GA, or non-diffusible signals, such as peptide ligands, are secreted from the embryo to ME (Figure 6B, red dashed arrows) and then stimulate gene expression in this tissue. However, the new finding about the TERF1 cascade and possible involvement of a PR- or wounding response in ME gene expression generated a new hypothesis of “mechanosensing.” In this hypothesis, pressure, rather than chemical molecules, which is generated by the embryo and placed onto ME cells (Figure 6B, blue arrow), triggers a wound response, *TERF1* expression, and then induction of the downstream genes in ME.

### The “touch” genes in arabidopsis seeds

**Figure 7:**
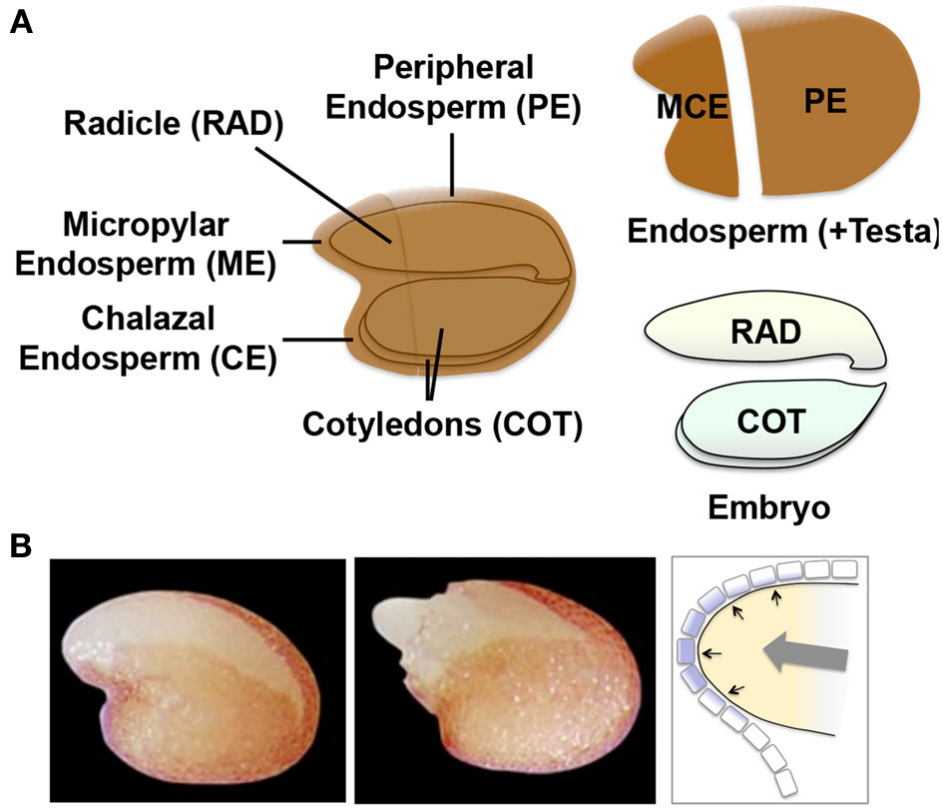
**Arabidopsis seed tissues and regulatory mechanisms of endosperm weakening. (A)** Schematic representation of the four different tissue parts of Arabidopsis seeds used for the transcriptome analysis (Dekkers et al., 2013). The micropylar (ME) plus charazal (CE) endosperm (MCE), peripheral endosperm (PE, similar to LE), radicle (RAD) and cotyledons (COT) are indicated in the scheme. **(B)** Photographs of Arabidopsis seeds at testa rupture (TR, left) and endosperm rupture (ER, right). Schematic representation of ME is shown to the right with the growth potential of the embryo (gray arrow) and pressure (black arrows) placed onto the single cell layer of endosperm (with purple filling).

A similar but more comprehensive and dynamic transcriptomic analysis in Arabidopsis seeds provided supporting evidence for the mechanosensing hypothesis. It is technically difficult to dissect ME from Arabidopsis seeds. Therefore, in this study gene expression was compared for the micropylar and charazal endosperm (MCE), peripheral endosperm (PE, similar to LE), radicle (RAD), and cotyledons (COT) (Dekkers et al., 2013) (Figure 7A). The high-resolution data set included many time points including those before and after testa rupture (TR) and endosperm rupture (ER), which are the signature events during germination and at the completion of germination, respectively (Figure 7B). This study demonstrated that TR was marked by activation of the specific genes in MCE, such as *TOUCH3* and *TOUCH4*, which are known to be induced by touch or thigmotropism (Braam, 2005). The comparison of MCE genes at TR in Arabidopsis seeds with the genes up-regulated by touching the aerial part of Arabidopsis plants (Lee et al., 2005) showed significant overlaps. These results suggest that ME gene induction in Arabidopsis seeds is also caused by touch or mechanosensing (Dekkers et al., 2013).

No conclusive evidence has been obtained to date for the mechanosensing or touch hypothesis. However, the new findings have great potential to re-draw the traditional view of ME gene regulation, which is a core mechanism of germination. It is well known that GA stimulates ME gene expression in the GA-deficient *gib-1* tomato seeds, which absolutely require GA for radicle emergence (Groot and Karssen, 1987; Nonogaki et al., 2000). The GA requirement for ME gene expression can be substituted by co-incubation of ME with the embryonic axes, suggesting that the embryo produces GA and secretes it to the endosperm (Groot and Karssen, 1987). There seems to be no doubt that ME gene expression is under the control of GA and the embryo. However, it should be noted that exogenous GA stimulates gene expression in both ME and LE when tomato seeds are dissected, while only ME is responsive when GA is applied to intact seeds (Martinez-Andujar et al., 2012). This raises the question as to why LE in an intact seed remains unaffected by GA or why only ME is responsive to it? The new hypothesis (mechanosensing or touch) could answer these questions. If the GA-dependent embryonic effects on ME gene expression are not directly exerted through chemical secretion but are indirectly mediated by the pressure provided by the radicle tip, the highly localized gene expression in ME, which is in close contact with the radicle tip, could be explained. Since generation of embryo growth potential, which causes the pressure onto ME, is dependent on GA (Ni and Bradford, 1993; Yamaguchi et al., 2001), the concept of pressure-triggered stimulation of ME gene expression is well integrated with the traditional concept (and evidence) of GA- and embryo dependency of ME gene expression. While the possibility of direct stimulation of ME by GA or insoluble secondary messengers should not be excluded, the recent data sets provided the new concept for embryo-endosperm interaction and opened the next phase of seed germination research.

## Perspectives for basic research and knowledge translation

### More discoveries expected through epigenetic study

**Figure 8:**
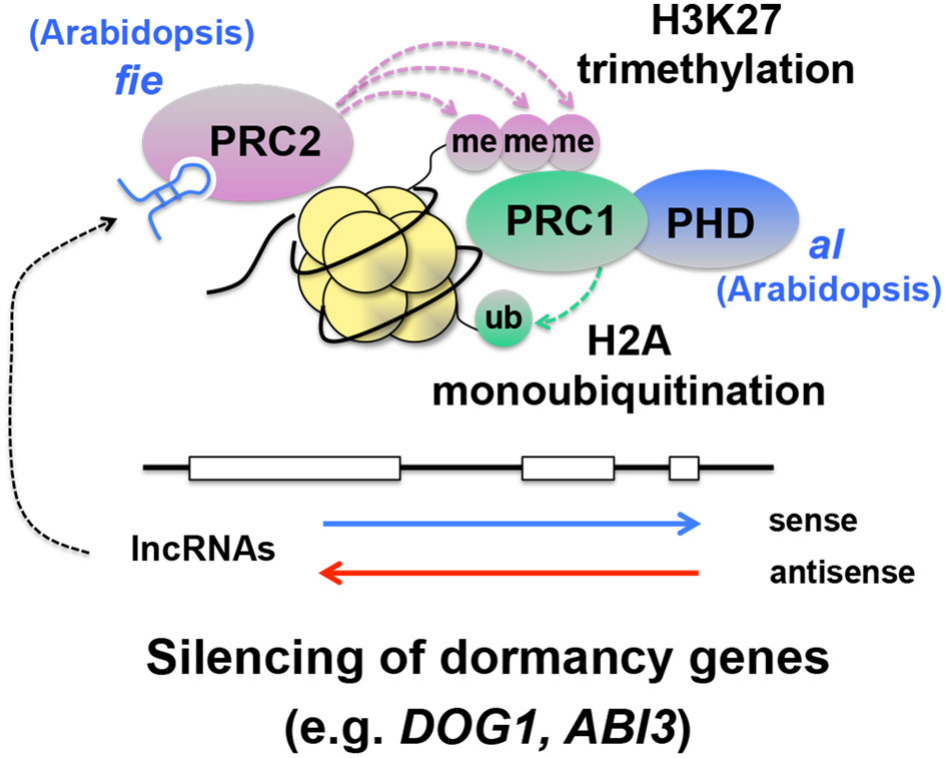
**Hypothetical integration of the known lncRNA-PRC pathway into the silencing mechanisms of seed dormancy genes**. In this scheme, long non-coding RNAs (lncRNAs) (Swiezewski et al., 2009; Heo and Sung, 2011), interact with Polycomb Repressive Complex 2 (PRC2), which causes histone 3 lysine 27 trimethylation (H3K27me3) (Simon and Kingston, 2009; De Lucia and Dean, 2011). This histone modification recruits PRC1, which monoubiquitinates H2A (Simon and Kingston, 2009). While H2B monoubiquitination promotes transcription elongation (see Figure 2), H2A monoubiquitination is thought to be a repressive mark and silence genes (Simon and Kingston, 2009). A mutation in *FERTILIZATION INDEPENDENT ENDOSPERM*(*FIE*), an essential component of PRC2, causes enhanced dormancy (Bouyer et al., 2011), supporting the idea that PRC suppresses dormancy genes and promotes germination. A mutation in ALFIN1-like (AL), a Plant Homeo Domain (PHD) finger that interacts with PRC1, also promotes dormancy (Molitor et al., 2014). Evidence has not been obtained for the involvement of specific lncRNAs in suppression of dormancy genes through PRC.

A number of discoveries were made in the recent studies of seed dormancy and germination. More significant discoveries will probably be made from epigenetic studies of seed dormancy and germination over the next few years. While bioinformatics and systems biology could generate new hypotheses, the exciting discoveries happening from characterization of seed dormancy mutants look very convincing and promising. Exploring these emerging mechanisms with forward genetics and biochemical and molecular approaches will result in more progress in seed dormancy research. The information obtained from individual mutants of chromatin remodeling was assembled into several schemes in this article to provide an overview of the frontier of this field. However, information to connect each component precisely in the schemes is still missing. For example, while histone methylation and subsequent silencing of *DOG1* by DNA methylation seems likely, contribution of DCL3, AGO4, and RdDM to the *DOG1*-dependent dormancy pathway is not clear (Figure 4). It is possible that siRNAs and long non-coding RNAs (lncRNAs), including antisense transcripts (Yamada et al., 2003; Liu et al., 2010; Sun et al., 2013), are involved in repression of key dormancy genes. Recent studies suggest that the Polycomb Repressive Complex (PRC), which is involved in histone methylation and gene silencing, also targets *DOG1* (Bouyer et al., 2011; Muller et al., 2012; Molitor et al., 2014). This is very interesting because PRC is known to mediate gene silencing triggered by expression of long non-coding RNA, at least in the case of the flowering gene *FLOWERING LOCUS C* (Swiezewski et al., 2009; De Lucia and Dean, 2011; Heo and Sung, 2011). It is possible that some dormancy genes are regulated through the lncRNA-PRC pathway (Figure 8), which could maintain dormancy genes “dormant.” Missing information in the current schemes of regulatory mechanisms of seed dormancy and germination genes might already be emerging from other epigenetic studies. In addition, the current schemes, which seem to be separate pathways, could be combined with each other and integrated into a single comprehensive scheme, through more discoveries. The crosstalk between the histone deacetylation and DNA methylation pathways is known (To et al., 2011; Kim et al., 2012), however little is known about their interaction directly linked to the seed dormancy mechanisms. This might be one of the areas in which the major discoveries could be made in the future.

### Knowledge translation of seed hormone biology

The topic of hormonal regulation of seed dormancy, such as the regulation of ABA or GA biosynthesis and deactivation enzymes by the environmental signals (e.g., light and temperature), was minimized in the discussion above, because it is well summarized elsewhere (Finkelstein et al., 2008; Seo et al., 2009) and this article focuses on emerging mechanisms and new hypotheses. Nonetheless, this is probably the area of seed biology that has been most advanced in the last decade, and from a knowledge translation point of view, this area has the greatest potential for agricultural application. For example, identification of the rate-limiting ABA biosynthesis gene *NCED* advanced our understanding of thermoinhibition of lettuce seed germination, which is a critical issue in agriculture. Now, we understand that thermoinhibition of germination at high temperature, which could induce secondary dormancy, is caused by *NCED* expression (Argyris et al., 2008, 2011). Likewise, screening of wheat populations for mutations in ABA 8'-hydroxyase, an ABA deactivation enzyme, has successfully identified the genetic lines, which are potentially resistant to PHS, another serious issue in agriculture (Chono et al., 2013). A separate screen for a mutation in the *ENHANCED RESPONSE to ABA* (*ERA*) gene also isolated PHS-resistant wheat lines (Schramm et al., 2013). The information about *MOTHER OF FT AND TFL1* (*MFT*) gene, which is a recently identified member of the ABA and GA signaling pathways in Arabidopsis (Xi et al., 2010), has already been translated into wheat (Nakamura et al., 2011; Lei et al., 2013; Liu et al., 2013).

**Figure 9:**
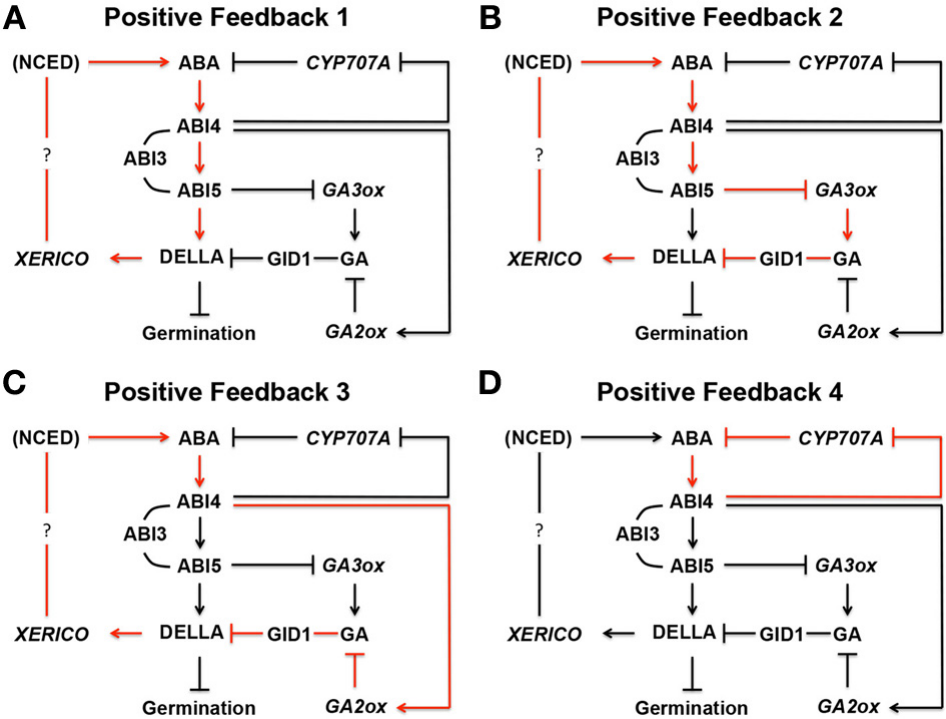
**Positive feedback loops in ABA biosynthesis in seeds. (A)** In Positive Feedback 1, ABA produced by NCED, a rate-limiting ABA biosynthesis enzyme, induces ABIs. ABI3, and ABI5 interacts with each other while ABI4 induces ABI5 by binding its promoter region. ABI5 binds to the promoter region of a *DELLA* gene, such as *RGL2*, and up-regulates its expression. DELLA then promotes expression of *XERICO*, which increases ABA biosynthesis through unknown mechanism(s). In this way, the originally produced ABA in seeds enhances ABA biosynthesis through positive feedback. **(B)** In Positive Feedback 2, ABI5 down-regulates *GA3ox*, a GA biosynthesis gene, and reduces GA and GA response by GID1, a GA receptor. Reduced GA levels stabilize DELLA protein, such as RGL2, and increases ABA biosynthesis through *XERICO*, as described above. **(C)** In Positive Feedback 3, ABI4 up-regulates *GA2ox*, a GA deactivation gene, resulting in the same outcome as Positive Feedback 2. **(D)** ABI4 down regulates *CYP707A*, an ABA deactivation gene. Therefore, ABA starts to accumulate in seeds, which further enhances the same pathway through positive feedback. In these schemes, many other components, which may be participating in the pathways, and negative feedback loops are omitted. ABI, ABA INSENSITIVE; *CYP707A, CYTOCHROME P450 707A*; DELLA, D (aspartic acid) E (glutamic acid) L (leucine) L (leucine) A (alanine) protein; GA, gibberellin; GA2ox; GA 2-oxidase; GA3ox, GA 3-oxidase; GID1, GA INSENSITIVE DWARF; NCED, nine-*cis*-epoxycarotenoid dioxygenase; RGA, REPRESSOR OF GAI; RGL2, RGA-LIKE 2; XERICO, “XERICO” (Greek for drought tolerant). The schemes are based on Ko et al. (2006), Zentella et al. (2007), Ariizumi et al. (2008), Piskurewicz et al. (2008), Bossi et al. (2009), Lee et al. (2012b), Cantoro et al. (2013), Kong et al. (2013), Lim et al. (2013), and Shu et al. (2013).

More progressive efforts are being made to translate seed hormone biology. It has been demonstrated that direct manipulation of the rate-limiting enzymes in the hormone metabolism pathways can successfully be used to alter seed performance. Silencing *NCED* with RNA interference can promote germination in lettuce seeds (Huo et al., 2013). In contrast, chemical induction of *NCED*, a single gene, was sufficient to suppress precocious germination in Arabidopsis, which can also be applied to PHS prevention in cereal crops (Martinez-Andujar et al., 2011). While the latter approach was tested in the model system Arabidopsis, the gene induction experiments in this study were performed with the chemical ligand that has been approved for field application by the U.S. Environmental Protection Agency, making the principle applicable to agriculture. Even more advanced system of *NECD* enhancement, which does not require chemical application, has been established recently, using a positive feedback mechanism. In this system, a chimeric *NCED* gene, which is designed to trigger positive feedback regulation, amplifies ABA biosynthesis and signaling in seeds and causes hyperdormancy in a spontaneous manner (Nonogaki et al., 2014). This positive feedback system was created based on the mechanisms emerged from, and the comprehensive understanding established by, the past research on the ABA metabolism and signaling pathway in seeds. The translational research unexpectedly revealed that a positive feedback mechanism is also present in the native system of *NCED* expression in seeds (Nonogaki et al., 2014), demonstrating the synergy between basic and translational research. Other positive feedback mechanisms in the hormonal regulation of seed dormancy and germination are also emerging from on-going discoveries (summarized in Figure 9). More findings and understanding of elegant pathways in nature will provide greater opportunities of knowledge translation, another frontier of research that should be expanded in the future.

### Conflict of interest statement

A patent application has been filed for a technology described in this article. The author declares that the research was conducted in the absence of any commercial or financial relationships that could be construed as a potential conflict of interest.
